# Therapeutic intraspinal stimulation to generate activity and promote long-term recovery

**DOI:** 10.3389/fnins.2014.00021

**Published:** 2014-02-27

**Authors:** Sarah E. Mondello, Michael R. Kasten, Philip J. Horner, Chet T. Moritz

**Affiliations:** ^1^Department of Rehabilitation Medicine, University of WashingtonSeattle, WA, USA; ^2^Department of Neurological Surgery, University of WashingtonSeattle, WA, USA; ^3^Center for Sensorimotor Neural Engineering, University of WashingtonSeattle, WA, USA; ^4^Graduate Program in Neurobiology and Behavior, University of WashingtonSeattle, WA, USA; ^5^Department of Physiology and Biophysics, University of WashingtonSeattle, WA, USA

**Keywords:** spinal cord injury, electrical activity, regenerative stimulation, neuroprosthesis, ISMS

## Abstract

Neuroprosthetic approaches have tremendous potential for the treatment of injuries to the brain and spinal cord by inducing appropriate neural activity in otherwise disordered circuits. Substantial work has demonstrated that stimulation applied to both the central and peripheral nervous system leads to immediate and in some cases sustained benefits after injury. Here we focus on cervical intraspinal microstimulation (ISMS) as a promising method of activating the spinal cord distal to an injury site, either to directly produce movements or more intriguingly to improve subsequent volitional control of the paretic extremities. Incomplete injuries to the spinal cord are the most commonly observed in human patients, and these injuries spare neural tissue bypassing the lesion that could be influenced by neural devices to promote recovery of function. In fact, recent results have demonstrated that therapeutic ISMS leads to modest but sustained improvements in forelimb function after an incomplete spinal cord injury (SCI). This therapeutic spinal stimulation may promote long-term recovery of function by providing the necessary electrical activity needed for neuron survival, axon growth, and synaptic stability.

## Cervical intraspinal microstimulation

Artificial stimulation via electrodes placed within the spinal cord parenchyma, termed intraspinal microstimulation (ISMS), is a promising technique for activating the spinal cord distal to an injury. ISMS may confer dual benefits of both re-animation of paralyzed limbs, as well as promoting plasticity leading to long-term recovery of function that outlasts the stimulation. Pioneering work demonstrated that intraspinal stimulation (ISMS) of the lumbar spinal cord is capable of directly evoking a range of hindlimb movements (Giszter et al., 1993; Mushahwar and Horch, 1998; Lemay and Grill, 2004). These movements often occur in functional synergies, or activate reflex circuits to produce complex movements (Tresch and Bizzi, 1999; Mushahwar et al., 2002).

**Figure 1:**
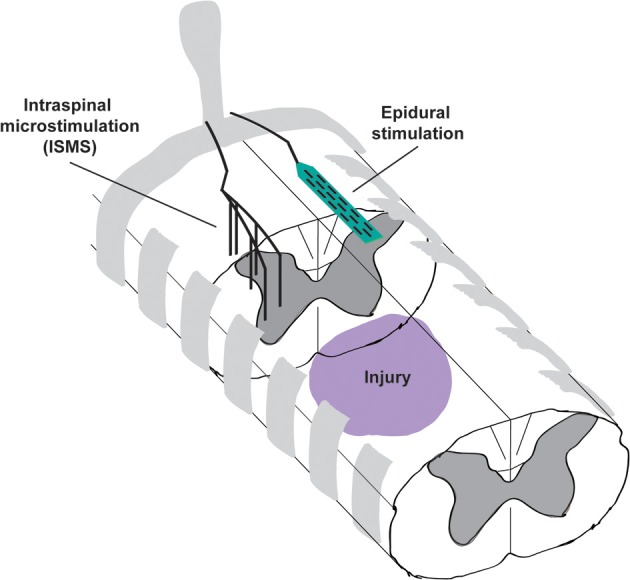
**Schematic illustrating the placement of intraspinal microstimulation (ISMS) electrodes and epidural stimulation electrodes caudal to a spinal cord injury.** ISMS electrodes penetrate the spinal cord to target the intermediate and ventral gray matter for activation of local spinal circuits. Epidural stimulation electrodes reside on the dorsal surface of the spinal cord, most likely activating the dorsal root entry zones or dorsal columns.

Recent work from our group and others explores the potential for cervical intraspinal stimulation to restore hand and arm movements (Moritz et al., 2007; Zimmermann et al., 2011; Sunshine et al., 2013). Cervical ISMS evokes a rich variety of forelimb movement both before and after injury (Sunshine et al., 2013), and also confers modest but sustained improvements in forelimb function that persist beyond the period of stimulation (Kasten et al., 2013). In this perspective we summarize the ability of intraspinal stimulation to both directly evoke movements and to promote long-term recovery. By contrast, we compare results from studies where stimulation is applied to the dorsal surface of the spinal cord, termed epidural stimulation (Figure 1). We focus on cervical intraspinal stimulation and highlight its potential for restoring critical movement to the hands and arms, both directly and via re-regulation of spinal circuits surrounding an injury.

### The pressing need for treatment of cervical spinal cord injuries

Among individuals with spinal cord injury (SCI), incomplete injury to the cervical spinal cord is the most common diagnosis (NSCISC, 2013). Restoration of hand and arm function is the highest treatment priority for individuals with cervical spinal cord injuries, *5-fold more important* than restoration of other functions lost to paralysis (Anderson, 2004). Despite this high priority, there are very few treatment options to bypass or repair the injured cervical spinal cord. Notable functional improvements have been demonstrated using combinatorial treatment strategies (Houle et al., 2006) or motor rehabilitation (Wang et al., 2011) following cervical hemisection or dorsal column lesion, respectively. The majority of clinical cases, however, are more complex contusion injuries with radiating secondary injury, demyelination and progressive axonal pathologies (Mctigue et al., 1998). Incomplete spinal cord injuries offer the distinct treatment advantage of axons that bypass the injury site and may not require long distance regeneration for restoration of function. This spared tissue could provide the crucial substrate for repair of conduction if it can be co-opted to transmit functionally useful signals around the injury.

### Leveraging intraspinal circuits for recovery from incomplete lesions

There is promising evidence for partial recovery following incomplete injuries that highlights the substantial capacity for plasticity of intraspinal circuits. For example, spinal interneurons sprout and form functional synaptic connections with motor neurons across a midline transection injury (Fenrich and Rose, 2009). Additionally, damaged corticospinal tract axons sprout above a dorsal hemisection forming new synaptic connections with propriospinal neurons that bypassed the lesion site (Bareyre et al., 2004). The functionality of this new circuitry was confirmed electrophysiologically and via improvement in locomotion. Indeed, humans with chronic, moderate incomplete SCI have also demonstrated substantial recovery of function following intensive motor retraining (Harkema et al., 2011) that can be maintained for several years (Behrman et al., 2008; Fox et al., 2010). These results highlight the capacity for rewiring of spinal circuits around an incomplete lesion, especially in younger subjects with perhaps more flexible synaptic connections (Pizzorusso et al., 2002).

With the substantial capacity of the spinal circuitry to remodel after incomplete injury, an underappreciated application of ISMS may be to promote or guide this plasticity by providing the necessary electrical activity needed for recovery of these orphaned spinal circuits. In the following section we review the substantial evidence demonstrating the importance of electrical activity for the recovery of neural networks after injury.

## Importance of activity for neural network recovery

Neuronal activity is an important regulator of multiple components of the nervous system, including the critical barriers to neuronal survival, differentiation, axonal growth, and synaptogenesis (For reviews see Spitzer, 2006; Borodinsky et al., 2012; Morimoto et al., 2012). The influential role of activity begins during the early stages of development and continues in the mature nervous system. This section describes the benefits of electrical activity with the goal of promoting recovery of the adult central nervous system after injury.

### Neural activity enhances survival and differentiation

Numerous data suggest that activity increases neuronal survival. For example, chronic electrical stimulation leads to increased survival of spiral ganglion cells after the application of a highly excitotoxic drug (Lousteau, 1987; Hartshorn et al., 1991). Multiple potential mechanisms may underlie this neuroprotective effect. The expression of a subset of genes that make neurons more resistant to stressful conditions, the activity-regulated inhibitors of death genes, are upregulated by synaptic activity, as detected in hippocampal neurons (Zhang et al., 2007; Tan et al., 2012). Furthermore, there is activity-regulated coupling between vascular cells and neurons critical for maintaining neuronal health and survival. For instance, numerous types of cortical neurons have shown that as they become more active, the blood flow and permeability of the blood brain barrier increases (for review see Leybaert, 2005) leading to greater delivery of oxygen, glucose, and other critical energy substrates (Cox et al., 1993; Harder et al., 1998; Iadecola and Nedergaard, 2007). The upregulation of brain derived neurotrophic factor (BDNF) is also enhanced by electrical activity (Al-Majed et al., 2000a). BDNF both increases spinal motoneuron synaptic plasticity (Al-Majed et al., 2000b, 2004), and protects neurons against oxidative DNA damage-induced death, as shown in cortical neurons (Yang et al., 2013). Several cyclic adenosine monophosphate (cAMP)-dependent mechanisms are similarly upregulated by activity and increase cell survival and axonal growth in retinal ganglion cells (Shen et al., 1999; Goldberg et al., 2002). Similar cAMP-dependent mechanisms enhance myelination in dorsal root ganglion cells (Malone et al., 2013). These effects are not necessarily restricted to just those neurons that are activated; activity-dependent neuroprotection has been shown to extend to regions beyond the field of neural activity in spiral ganglion cells (Leake et al., 1991). Thus, increasing the survival and participation of viable neuronal populations could lead to the formation of a circuit capable of bypassing an incomplete lesion and restoring a greater variety of motor and sensory functions.

### Axonal growth and elongation are positively regulated by neural activity

Axonal growth may be critical for the formation of new circuits after SCI, and is also largely regulated by activity. *In vitro*, acetylcholine and glutamate attract growth cones into specific directions in a calcium dependent manner (Zheng et al., 1996). Furthermore, motoneurons from a mouse model of spinal muscular atrophy with reduced spontaneous calcium transients exhibit reduced axonal growth and growth cone size (Jablonka et al., 2007). Other studies show that the frequency of rhythmic bursting in the embryonic spinal cord affects the dorso-ventral and antero-posterior path-finding of developing axons (Hanson and Landmesser, 2006). In the adult nervous system, exogenously induced electrical activity increases the expression of the axon-growth associated gene GAP-43 in axotomized sensory and motor neurons, which in turn leads to the increased speed and accuracy of motor axon regeneration and reinnervation (Al-Majed et al., 2000a). Additionally, acute stimulation of ventral spinal cord cell transplants following sciatic nerve denervation leads to an increase in the number of myelinated axons and functionally innervated muscles (Grumbles et al., 2013). These studies suggest that inducing activation of spinal circuitry after injury may significantly increase axonal growth and regeneration, as well as the specificity of the circuit itself, leading to greater functional recovery.

### Neural activity induces synaptogenesis and dendrite stability

Synaptogenesis and novel dendrite stabilization is necessary for functionally restorative network formation after SCI. Activity is known to strongly regulate dendrite stabilization during development. For example, developing dendrites stabilize in response to cholinergic neurotransmission and local calcium-induced calcium release in the early stages of synapse formation (Lohmann et al., 2002). Furthermore, during the developmental phenomena of synaptic competition, synapses that are the most proficient at inducing postsynaptic activity remain viable while ineffective synapses are permanently removed (Balice-Gordon and Lichtman, 1994; Buffelli et al., 2003). The recruitment of progranulin to synapses along axons, which promotes synapse formation (Tapia et al., 2011; Petkau et al., 2012) increases in the presence of neuronal activity, indicating an activity-dependent regulation of synapse number and structure (Petoukhov et al., 2013). Enhancing the activity of neural circuits caudal to the injured spinal cord may both increase synaptogenesis, and act to stabilize new circuitry once it is formed.

Taken together, these studies strongly demonstrate that neural activity is necessary to promote long-term recovery of functional circuits after SCI. Intraspinal stimulation is one potential method for introducing this activity, either with the goal of directly re-animating the limbs, or perhaps ideally to promote sustained recovery of function.

## Evidence for benefit of electrical stimulation after spinal cord injury

### Direct benefits of spinal stimulation

#### Intraspinal microstimulation (ISMS) of the cervical spinal cord

Stimulation within the spinal cord activates local spinal networks in specific patterns to evoke functional movements. Building on extensive work demonstrating the ability of ISMS within the lumbar spinal cord to evoke standing and stepping movements (Mushahwar and Horch, 1998; Mushahwar et al., 2002, 2004), we have demonstrated that forelimb movements can also be evoked by ISMS delivered to the cervical spinal cord in non-human primates (Moritz et al., 2007). Zimmermann et al. (2011) then elegantly demonstrated that a reach and grasp movement could be re-animated by combining stimulation of multiple locations within the spinal cord of the anesthetized primate.

More recently we explored the forelimb movements evoked by cervical ISMS both before and after injury in a clinically-relevant rodent model of mid-cervical contusion injury (Sunshine et al., 2013). A wide variety of somatotopically organized forelimb movements could be evoked prior to injury from throughout the cervical spinal cord. Following injury, there was a transient period lasting approximately 3 weeks where movement variety was dramatically reduced. By 6 and 9 weeks after injury, however, a variety of movements could again be evoked via cervical ISMS, which were not statistically different from the effects prior to injury (Sunshine et al., 2013).

#### Electrical stimulation after spinal cord injury improves motor function

Electrical stimulation applied to the epidural surface of the spinal cord shows great promise for directly improving motor function after SCI. Epidural stimulation of the lumbar spinal cord can facilitate stepping movements after complete spinal transection, especially when combined with serotonin agonists and movement of a motorized treadmill underfoot (Ichiyama et al., 2005; Gerasimenko et al., 2008; Edgerton and Harkema, 2011). When combined with robotic assistance and motor training over complex surfaces, epidural stimulation also appears to facilitate the formation of a relay circuit bypassing a complex, staggered lesion in animal models (Courtine et al., 2008; Van Den Brand et al., 2012). In addition, an individual with American Spinal Injury Association (ASIA) grade B incomplete injury was able to volitionally move his legs and support body weight during the activation of surgically-implanted epidural stimulation (Harkema et al., 2011). While these examples are inspiring, with the exception of important autonomic functions, improvements in skeletal motor function did not persist beyond the period of stimulation in this landmark study. As described below, lasting improvements in spinal circuit function have been noted after stimulation delivered to the brain, muscles, and intraspinal locations after injury.

### Persistent benefits of electrical stimulation after injury

Electrical stimulation applied to the cortex or brainstem after selective SCI promotes axon sprouting (Brus-Ramer et al., 2007) and improves motor function persisting beyond the period of stimulation (Carmel et al., 2010). In addition, electrical stimulation of fiber tracts in the pyramids or spinal white matter promotes sprouting and maintenance of ipsilateral spinal connections that are otherwise pruned during development (Salimi and Martin, 2004). In an early case study using epidural stimulation applied to the lumbar spinal cord, an individual with incomplete (ASIA C) SCI improved functional walking, with some motor benefits persisting beyond the period of stimulation (Herman et al., 2002). Since new connections are formed spontaneously after injury but subsequently lost if they do not project below the lesion (Bareyre et al., 2004), intraspinal stimulation caudal to the injury may be key to creating and maintaining a network of connections bypassing an incomplete spinal injury.

Trains of electrical stimulation delivered to peripheral nerves have been shown to substantially enhance function of both motor and sensory axons following nerve transection and subsequent surgical repair (Brushart et al., 2002, 2005). Functional electrical stimulation (FES) applied to muscles may also promote plasticity and circuit re-organization (reviewed in Barbeau et al., 2002). FES applied to the hindlimb muscles after SCI provides lasting improvements in gait (Jung et al., 2009), and also reduced muscle tone and improved spasticity (Mirbagheri et al., 2002). Notably, we have observed substantial reductions in tone of the injured forelimb in rodents receiving long-duration intraspinal stimulation below the injury, which likely contributes to their improved functional abilities that persist beyond the period of stimulation (Kasten et al., 2013).

### Therapeutic benefits of ISMS

Therapeutic ISMS of the cervical spinal cord evokes complex and often highly-functional synergies, but we also have recent evidence that stimulating the spinal cord below an injury improves motor function with benefits lasting beyond the period of stimulation (Kasten et al., 2013). Animals received a lateralized contusion injury in spinal cord segment C4, and ISMS electrodes were implanted caudal to the lesion 3 weeks later in segments C6-C7 (Figure 1). Beginning 4 weeks after injury, animals received therapeutic intraspinal stimulation for 7 h/day, 5 days/week. Recovery was measured using the precision forelimb reaching task (Schrimsher and Reier, 1992; Mckenna and Whishaw, 1999), in all cases without the presence of stimulation.

**Figure 2:**
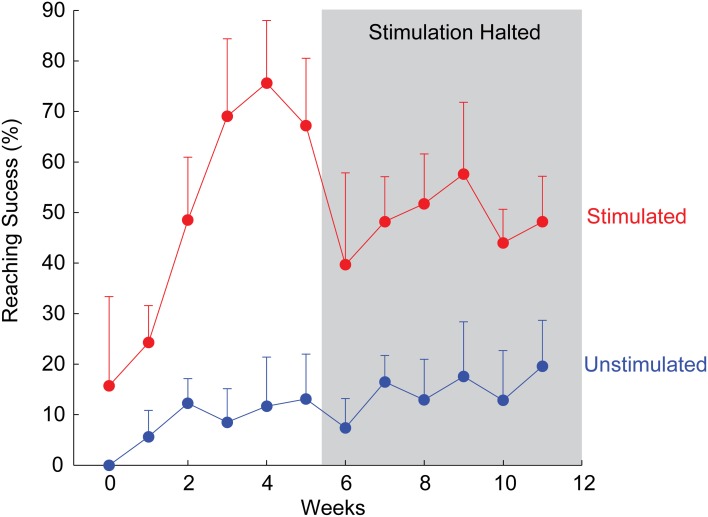
**Example of rapid recovery of forelimb reaching ability for one animal receiving therapeutic ISMS, compared to an unstimulated animal.** After 5 weeks, stimulation was halted due to implant failure, allowing examination of sustained recovery with no further stimulation. Mean + SD for each animal.

In a particularly interesting example, an animal in the stimulated group recovered to 75% of pre-injury reaching ability within just 4 weeks (Figure 2). Notably, stimulation in this animal was discontinued after week 5 due to implant failure, providing the opportunity to observe lasting recovery with no further stimulation. Although reaching scores reduced immediately upon halting daily stimulation, success rates stabilized well above pre-treatment levels and those of unstimulated animals (Figure 2), suggesting a long-term or therapeutic benefit from ISMS (Kasten et al., 2012).

Animals that continued to receive therapeutic ISMS for the remainder of the 12 weeks treatment period also significantly outperformed their unstimulated counterparts (Kasten et al., 2013). Detailed examination of the reaching components revealed the greatest improvements in the ability to aim and advance the arm in the precision forelimb reaching task, consistent with improved coordination and proprioception. Thus, therapeutic spinal stimulation appears to provide the necessary electrical activity missing after injury and leads to the re-regulation of neural circuits deprived of natural descending drive.

## Remaining challenges in therapeutic ISMS

While the applications of ISMS to promote activity and functional recovery is very promising, several technical hurdles must be overcome prior to widespread clinical adoption. The long-term stability of stimulating electrodes within the spinal cord must be demonstrated, as negative tissue responses are common following implantation of electrodes within the central nervous system (Shain et al., 2003; Spataro et al., 2005). Fortunately, delivery of stimulation may be less sensitive than recording of neural signals via implanted probes, and animal studies of ISMS electrode longevity demonstrate only modest tissue response (Mushahwar et al., 2000; Bamford et al., 2010) with only gradual increases in stimulation thresholds (Kasten et al., 2013). Additional hardware challenges can likely leverage the success of clinical epidural stimulation, currently approved as a treatment for chronic pain (Foreman and Linderoth, 2012). This existing platform provides examples of implantable stimulators and electrodes placed in the vicinity of the spinal cord with long-term efficacy.

To maximize the success of ISMS, the future depends upon the development of better insight in to the physiologic and molecular mechanisms of activity whereby axonal or circuit function is improved. In turn, complete restoration of neural function is likely to be achieved only by combining ISMS with complimentary therapies. Individual treatments rarely demonstrate large functional improvements, particularly in the chronic phase of SCI, and in clinically-relevant injury models. For example, combinations of intensive rehabilitation, pharmacological treatment and epidural stimulation show promise following a staggered hemisection injury (Van Den Brand et al., 2012; but cf. Slawińska et al., 2012). To effect recovery following chronic contusive injuries to the spinal cord, combinations of therapeutic spinal stimulation (epidural or ISMS) and additional modalities will likely be required. Examples of these additional interventions include pharmacological therapy (Musienko et al., 2011), application of stem cell therapy near the injury site (Parr et al., 2008; Nutt et al., 2013), treatments to minimize scar formation or encourage axonal outgrowth (Qiu et al., 2002; Pearse et al., 2004) and myelination (Lu et al., 2002). Intraspinal stimulation may collaborate with all of these approaches by creating the necessary activity to promote long-term repair of the injured spinal cord.

## Conclusion

Cervical ISMS holds tremendous potential for both directly re-animating forelimb movements as well as promoting long-term recovery of function after SCI. Building on substantial evidence for the importance of activity in nervous system development and recovery, therapeutic ISMS may provide the otherwise absent electrical activity needed for neuron survival, axon growth, and synaptic stability. The application of therapeutic stimulation to the spinal cord distal to a lesion may therefore be critical in promoting repair of spinal circuits following injury, either alone or in combination with other rehabilitative and pharmacological interventions.

### Conflict of interest statement

The authors declare that the research was conducted in the absence of any commercial or financial relationships that could be construed as a potential conflict of interest.
